# Preventing Emerging and Re-emerging Infections in the Eastern Mediterranean Region: Gaps, Challenges, and Priorities

**DOI:** 10.2196/14348

**Published:** 2019-10-09

**Authors:** Rawan Araj, Sultan Alqasrawi, Sahar Samy, Ghaya Alwahdanee, Jamal Wadi, Jawad Mofleh, Tarek Alsanouri

**Affiliations:** 1 Global Health Development/Eastern Mediterranean Public Health Network Amman Jordan; 2 Jordan Ministry of Health Amman Jordan; 3 Egypt Ministry of Health Cairo Egypt; 4 The Medical School University of Jordan Amman Jordan

**Keywords:** emerging, infectious, diseases, Eastern Mediterranean

## Abstract

**Background:**

The Eastern Mediterranean Public Health Network, supported by the Biosecurity Engagement Program, contributed significantly to strengthening the preparedness and response to the emerging and re-emerging infections in the region.

**Objective:**

This study aimed to determine the gaps, challenges, and priorities for preventing the emerging and re-emerging infections, with a focus on biosafety and biosecurity in four countries of the region, namely, Egypt, Iraq, Jordan, and Morocco.

**Methods:**

A total of two different methods were used to determine the gaps and priorities for preventing the emerging and re-emerging infections. The first method was a rapid assessment for the preparedness and response to the emerging and re-emerging infections in four countries of the region, with a focus on biosafety and biosecurity. The second method was a face-to-face round table meeting of the participating teams for two days, where the teams from all countries presented their countries’ profiles, findings, priorities, and gaps based on the countries’ assessments.

**Results:**

The assessment and meeting resulted in several priorities and recommendations for each of the countries in the areas of legislation and coordination, biosafety and biosecurity, surveillance and human resources, case management and response, infection control and prevention, and risk communication and laboratory capacity.

**Conclusions:**

Many recommendations were relatively consistent throughout, including improving communication or building collaborations to improve the overall health of the country.

## Introduction

### Background

The Eastern Mediterranean region is a hotspot for emerging and re-emerging infectious diseases [1]. Almost half of the countries in the region have witnessed epidemics from emerging infectious diseases over the past years [2]. Many outbreaks were detected, investigated, and contained in some countries of the region in the last few years, including yellow fever [3], Middle East respiratory syndrome [4], cholera [5], avian influenza A (H5N1) infection [6], dengue fever [7], and chikungunya [8-10]. These epidemics had a high burden on the health system and the socioeconomic development of the region.

The risk of the emerging and re-emerging infectious diseases has increased significantly in the past years owing to different factors, some of which are related to humanitarian emergencies, wars, fragile health systems, weak surveillance, and limited laboratory diagnostic capacity [11]. A high number of internally displaced persons and refugees living in overcrowded and overburdened spaces, with little or no access to basic health care services [12], has contributed to the emergence and reemergence of epidemic diseases in the region [13].

The regional preparedness, response, and control efforts face major challenges such as poor vector surveillance capacities, lack of integrated vector management approaches, weak multidisciplinary and intersectoral collaboration, and the absence of comprehensive preparedness and response plans.

Protecting people’s health by combating outbreaks remains a national priority for all countries in the region. This is also important to ensure maximum protection of the people against the international spread of these diseases and its role in global health security. A regional collective approach to address the emerging and re-emerging infections as a health security issue will permit the rapid detection of novel biological threats and assist in identifying innovative and locally crafted solutions for the management of these disease threats, both from a security and safety perspective as well as from a disease control standpoint.

### Objectives

The Eastern Mediterranean Public Health Network (EMPHNET), supported by the Biosecurity Engagement Program, contributed significantly to strengthening the preparedness and response to the emerging and re-emerging infections in the region. This study aimed to determine the gaps, challenges, and priorities for preventing the emerging and re-emerging infections, with a focus on biosafety and biosecurity in 4 countries of the region, namely, Egypt, Iraq, Jordan, and Morocco.

## Methods

### Gaps and Priorities Identification

A total of two different methods were used to determine the gaps and priorities for preventing the emerging and re-emerging infections in 2015. The first method was a rapid assessment for preparedness and response to the emerging and re-emerging infections in 4 countries of the region, with a focus on biosafety and biosecurity. The second method was a face-to-face round table meeting of the participating teams for two days, where the teams from all the countries presented their countries’ profiles, findings, priorities, and gaps based on the countries’ assessments.

### Rapid Assessment

The EMPHNET team developed a questionnaire to obtain up-to-date information on the status of the participating countries regarding preparedness and other capacities required to detect and respond to the outbreaks of the emerging and re-emerging infections. The team comprised experts in emerging and re-emerging infections, biosafety and biosecurity, outbreak investigation, and response. The questionnaire tackled the following areas: legislation and coordination (4 questions), biosafety and biosecurity (13 questions), surveillance and human resources (12 questions), case management and response (2 questions), infection control and prevention (7 questions), risk communication (4 questions), and laboratory capacity (4 questions). The questionnaire included yes, no, remarks and percentage for responses and a section for listing the priorities and gaps for urgent consideration. It was structured as Web-based link that was sent to the focal points of each participating county. The focal points met with their respective teams in their own countries and filled the questionnaire. The individuals included in the country teams that responded to the questionnaire were surveillance officers, animal health officers, officers from the Central Public Health Laboratory, International Health Regulations (IHR) focal points, communicable diseases control officers, and Field Epidemiology Training Program (FETP) coordinators.

### Round Table Meeting

A round table on *Preventing Emerging and Re-emerging Infections*, which aimed at improving the biorisk management and best practices in relation to disease control efforts for emerging pathogens, was conducted. The round table session was intended for the public health officials from the region as part of strengthening the preparedness and response to emerging and re-emerging infections. The organizing team invited director generals, senior surveillance officers, animal health officers, directors of the Central Public Health Laboratories, IHR focal points, director for communicable diseases, FETP coordinators, and senior officers of the United Nations Relief and Works Agency’s health section to participate in the round table meeting. In this round table meeting, the experts gave an overview of the region and provided some information on the main known threats to the region; then, the countries’ representatives presented their countries’ profiles and findings of the rapid assessments. The country teams discussed their findings and the countries’ strengths, gaps, weaknesses, and recommendations. The representatives, delegates, and facilitators of all countries came up with areas of priority for immediate consideration and areas for short- or long-term consideration. Finally, they recommended several short-, medium-, and long-term activities to improve the preparedness and response to the emerging and re-emerging infectious diseases’ threats at the subregional level. At the round table sessions, special attention was given to biosafety, biosecurity, and biorisk management issues related to the emerging and re-emerging infections. The organizing team transcribed discussions, comments, questions, and answers throughout the round table.

## Results

### Responses of Countries

On the basis of the rapid assessment responses and the round table discussions of the representatives from Egypt, Iraq, Jordan, and Morocco, the major gaps, challenges, and priorities for the different focus areas are discussed below.

### Legislation and Coordination

The countries’ representatives reported that they have a public health law that allows for the detection and investigation of the emerging and re-emerging diseases’ cases and outbreaks, an assigned high-level intersectoral coordination body at the national level, and a functional emergency operations center at the central level. Overall, 3 countries confirmed that they have a biorisk management plan and that they update their plans regularly. Moreover, 3 countries reported that they regularly conduct risk assessment of major health facilities at the national level. Morocco reported that they do not have a biorisk management plan, and Iraq reported that they do not have mechanisms to detect any violation of the biorisk management plan. Iraq also reported that they do not conduct regular risk assessment of major health facilities.

### Biosafety and Biosecurity

All countries reported that they have logbooks to record toxins and biological agents where necessary, measures are in place to identify and manage risk associated with general safety, staff are handling dangerous substances with enough care, and their general surveillance systems are based on proper risk assessment. In addition, they reported that they have enough controls in place for the physical security of infectious materials of all kinds commensurate with the assessed risks.

### Surveillance and Human Resources

Countries reported that they have comprehensive public health surveillance systems, and 2 out of the 4 countries reported that they have early warning and response surveillance systems as well. The surveillance system in Jordan reports on weekly and monthly basis, but outbreaks are reported immediately. The frequency of reporting of surveillance sites in Iraq, Morocco, and Egypt are immediate and daily for notifiable diseases and weekly and monthly for other diseases. In addition, all countries reported that the public health surveillance systems are linked with animal health surveillance systems. Furthermore, 3 out of the 4 countries reported that their public health surveillance systems are linked with response units and the same number reported that they have rapid response teams at the provincial and governorate levels. Iraq reported that the surveillance system is not linked with response teams, and Morocco reported that they do not have rapid response teams at the provincial and governorate levels. All countries reported that they have specific investigation, response, and medical teams for the management of emerging and re-emerging infections, which have capacities for contact tracing, isolation, and quarantine. Furthermore, 3 countries revealed that they have a regular feedback system to health facilities, whereas Jordan reported informal feedback system.

### Case Management and Response

Only 2 countries reported that they have generic protocols for the treatment of the emerging and re-emerging infections. Countries lacking these protocols were Morocco and Egypt. All countries reported that they have updated protocols for the sample collection of the emerging and re-emerging infections. A total of 3 countries reported that they completed the risk assessment of main laboratories; in addition, the same number reported that the risk mitigation and control measures in the laboratories are in place, and they confirmed that the standard operating procedures (SOPs) for waste management in laboratories are practiced. Furthermore, they reported that they have strong links with the regional reference laboratories.

### Infection Control and Prevention

A total of 3 out of the 4 countries reported that they have a national infection prevention and control program. All countries reported that they have trained provincial infection control and prevention teams, a national policy for medical waste management, and enough personal protective equipment for general use and for the use of infection control and prevention teams. Morocco reported that they do not have a functional infection control and prevention program at the ministry level and that no entity regularly reports on the status of the national infection control and prevention goals and strategies.

### Risk Communication and Laboratory Capacity

A total of 2 out of the 4 countries reported that they have World Health Organization–certified (WHO-certified) point of entry, whereas Jordan and Egypt reported not having any WHO-certified point of entry. All participating countries reported that they have a national risk communication plan, well-known national spokesperson, and 2-way real-time communication with media.

The participants of the round table highlighted that the interdisciplinary coordination and communication within the health sector and throughout the board with other actors are weak. They also agreed that the responses to the events are not on time. They mentioned that the delay in the detection of first few cases (surveillance), sample collection, transportation and confirmation, and the limited technical resources and financial means are major challenges to provide a proper response to public health events. Participating representatives also reported that they face multidrug-resistant infections in their countries. In addition, they identified gaps in specimen packaging and shipment and raised concerns over public health surveillance and other capacities required for the points of entry and exist in all countries. Risk communication and involvement of media were identified to be weak. The media tend to publish unrealistic negative news more than the realities in the field, as reported.

The public health laboratories are functioning in Iraq but with limited diagnostic kits and limited laboratory supplies. Jordan’s team identified other problems, such as limited number of qualified trained personnel and high staff turnover rates, which lead to under reporting or incomplete reporting of surveillance data, biosafety events and incidents, financial shortfall for procurement of infection control materials, and financial shortfalls in implementing infection control measures. The main gaps identified by the Moroccan team included the lack of institutional link with animal health surveillance and the inadequate coordination to implement an integrated surveillance system for zoonotic diseases between the Ministry of Health and the Ministry of Agriculture (animal husbandry).

## Discussion

### Principal Findings

Representatives from the participating countries reported that they have surveillance systems, laboratories, and infection control and prevention programs in place, although these systems were not perfect and needed support in many areas. Strengthening of the surveillance systems by adding components such as the disease early warning system was one of the requests. The risk assessment and risk mitigation of main health facilities and laboratories should be conducted regularly. Bridges for communication and collaboration between animal and human health to detect, confirm, and respond to outbreaks of animal origin should be built or improved at the national levels. The limitation of these findings is that they may not reflect all or the most pressing gaps, challenges, and priorities of the countries.

Many recommendations were relatively consistent throughout; they included improving the communication or building collaborations to improve the overall health of the country. The general recommendations by countries can be summarized as follows:

- Improve coordination, communication, and interaction among partners, stakeholders, and donors and adopt a systematic approach based on the accepted national and regional guidelines and standards while dealing with emerging and re-emerging infections
- Establish outbreak management teams, and not only outbreak investigation teams, to improve timely and effective response
- Develop and agree on the detailed SOPs for operational issues and challenges
- Strengthen the links with mass media and user-friendly social media
- Improve and strengthen public health surveillance systems.

### Highlights by Country

The country-specific priorities and recommendations are presented in Textbox 1.

**Country-specific priorities and recommendations to support the prevention of emerging and re-emerging diseases.**

Egypt

- Review the surveillance and response system
- Improve and update public health surveillance guidelines
- Establish and improve electronic surveillance systems, for example, early warning system and event-based surveillance
- Develop general and generic guidelines and protocols for the management of emerging and re-emerging infections
- Improve biosafety and biosecurity through training and capacity building of concerned staff
- Technical and financial support to establish disease early warning system
- Technical and financial support to establish event-based surveillance and its related guidelines
- Build capacity through training
- Technical and financial support to prepare and devise national preparedness and response to outbreaks and pandemics

Iraq

- Stronger links between animal and human health surveillance systems
- Response strategies should include animal health authorities to implement better response measures while facing zoonotic diseases
- Specific trainings activities, for example, training of rapid response teams and refresher trainings, are required for better response to outbreaks and events
- Review, assessment, and evaluation of the infection control and prevention programs
- Improve connections and links with regional and global laboratory networks to improve the on-time detection and confirmation of diseases and improve the specimen transportation and delivery
- National communication and risk communication strategies should be developed
- Biorisk management plan should be developed, enforced, and implemented and a mechanism should be emplaced to detect any breach of the biorisk management plan and its related standard operating procedures
- Training of health care workers on biosafety and biosecurity and emerging and re-emerging infections
- Coordination meetings with the neighboring countries on the emerging and re-emerging infections such as Ebola
- Provision of laboratory diagnostic kits, training, and monetary motivation

Jordan

- Improve the awareness of decision and policy makers and obtain their commitment to improve available gaps
- Improve biorisk management (biosafety and biosecurity risk management) by a national plan implementation
- Strengthen infection control and prevention in hospitals
- Establishment of public health surveillance systems at the point of entries and improve the shipment of biological samples
- Improve laboratory capacity for the early detection of the emerging and re-emerging infections
- Training of health care workers on biorisk management (biosafety and biosecurity), emerging and re-emerging infections, and importance of surveillance and timely reporting

Morocco

- Update the existing public health law in Morocco
- Establish a multidisciplinary and multisectorial technical committee responsible for documenting, monitoring, and tracking the international epidemiological situations, conducting risk analysis, and prioritizing the emerging diseases that have a greater risk in Morocco
- Develop specific programs or guides to be able to act upon the emerging diseases
- Bridge gaps and improve communication between animal and human health services to pave road for establishing an animal/human health unit in the Ministry of Health
- Establish a national program with all components for infection prevention and control
- Improve surveillance system by updating the list of notifiable diseases
- Improve the laboratory-based surveillance system to monitor the activities of certain pathogens and improve the diagnostic capacity

- Conduct refresher training activities for health professionals
- Conduct seroprevalance studies to estimate the prevalence of diseases such as leptospirosis, brucellosis, West Nile fever, and dengue fever in Morocco
- Establishment of animal/human health unit (zoonotic diseases unit)
- Develop a national plan to prevent health care–associated nosocomial infections

## Abbreviations

EMPHNET: Eastern Mediterranean Public Health Network
FETP: Field Epidemiology Training Program
IHR: International Health Regulations
SOP: standard operating procedure
WHO: World Health Organization
